# Effects of Directionality on Interpreting Performance: Evidence From Interpreting Between Chinese and English by Trainee Interpreters

**DOI:** 10.3389/fpsyg.2021.781610

**Published:** 2021-11-26

**Authors:** Isabelle Chou, Kanglong Liu, Nan Zhao

**Affiliations:** ^1^School of Foreign Languages, University of Electronic Science and Technology of China, Chengdu, China; ^2^Department of Chinese and Bilingual Studies, The Hong Kong Polytechnic University, Kowloon, Hong Kong SAR, China; ^3^Department of Translation, Interpreting and Intercultural Studies, Hong Kong Baptist University, Kowloon, Hong Kong SAR, China

**Keywords:** directionality, trainee interpreters, English–Chinese interpreting, consecutive interpreting, psycholinguistics

## Abstract

Interpreters can either interpret from the first language (L1) to the second language (L), or in the other direction. Understanding translation and interpreting as a direction-dependent process contributes to a wider and more critical view regarding the role of both languages in the process, as well as the identity, perspectives, and preferences of translators. The effect of directionality primarily weighs on stimulus and individual factors. This study explores the impact of directionality on the performance of trainee interpreters by examining four critical aspects of quality in target speeches, namely: speech rate, information completeness, delivery, and quality of expression. We observed an advantage for L2-L1 over L1-L2 interpreting in the form of interpreting quality (i.e., delivery and quality of expression) but not in content (i.e., the level of information retained in the target language). These effects of interpreting directionality suggest an important role of L2 proficiency in interpreting. Moreover, L1-L2 interpreting is cognitively demanding compared to L2-L1 interpreting for trainee interpreters. This research sheds light on the cognitive mechanisms of interpreting in different directions and provides pedagogical recommendations for training interpreters.

## Introduction

Interpreting can differ in its directionality: it can be done from the interpreter’s first language (L1) into their second language (L2) or the other way around (i.e., L1-L2 or L2-L1 interpreting; Pavlović, 2007). With more insights gained from the critical cognitive approach to the translation research process, scholars have started to challenge the notion that translation and interpreting should only be conducted unidirectionally from speakers’ L2 to L1 (Ferreira and Schwieter, 2017). In order to address such an issue, researchers have taken an increasing interest in the role of directionality on interpreters’ performance and quality of interpreting. Some studies have found that interpreters perform better in L2-L1 interpreting than in the other direction (Seleskovitch, 1999), while others have reported contrasting evidence (Tommola et al., 2000; Van Dijk et al., 2011). A considerable number of studies revealed that the effect of directionality is closely linked to other factors including language-pair specificity (Bartłomiejczyk, 2006), level of L2 proficiency, and the encyclopedic knowledge of the translator/interpreter (Pokorn et al., 2020).

During the interpreting process, a plethora of factors can affect the performance of the interpreter and the quality of interpreting. This is particularly so in consecutive interpreting (CI) which is a cognitively demanding multitask involving different cognitive processes (e.g., comprehension, note-taking, memory maintenance, production). Therefore, it is of paramount difficulty to identify how directionality may serve as a modulator in CI. Previous studies found that directionality has some impact on lexical translation with an asymmetry effect, where L2-L1 translation or interpreting is more fluent than the reverse direction (Kroll and Stewart, 1994; Sholl et al., 1995; Chmiel, 2016; Lin et al., 2018). The translation asymmetry effect is found to decrease in lexical translation when participant’s L2 proficiency increases (De Groot, 1992; García, 2015), which shows that L2 proficiency is a confounding variable in this regard. However, whether the translation asymmetry effect holds true at the discourse level has not been thoroughly investigated, particularly with student interpreters. Though some studies have attempted to explore the asymmetry effect in CI or SI, most of them have largely been confined to one isolated indicator of interpreter’s performance or interpreting quality, such as fluency (Lin et al., 2018; Yuan and Wang, 2019), accuracy (Chen, 2020), and interpreters’ encyclopedic knowledge (Pokorn et al., 2020). As isolated indicator can only tell one aspect of the interpreting products, it is of significant importance to adopt a multi-dimensional approach to study directionality effect so that a more detailed picture can be obtained.

Most CI or SI studies that go beyond lexical translation are confined to certain aspects of interpreters’ performance or interpreting quality, such as fluency (Lin et al., 2018; Yuan and Wang, 2019), accuracy (Chen, 2020), and interpreters’ encyclopedic knowledge (Pokorn et al., 2020). No study has yet extended the inquiry of the directionality effect from lexical processing to multi-dimensional interpreting performance. To bridge this gap, the present study aims to explore the mechanisms by which directionality may impact the interpreting quality and performance of interpreters with L1 in Chinese and L2 in English.

## Directionality in Translation and Interpreting Studies

### Revised Hierarchical Model and Directionality

As one of the established models in bilingual language processing, the revised hierarchical model (RHM) of bilingual language processing (Kroll and Stewart, 1994) proposes asymmetric links between L1-L2 and L2-L1 translation equivalents. Bilinguals have two language-specific lexicons and a common conceptual store. Specifically, L1 and L2 translation equivalents are separately stored in the language-specific lexicons but share similar semantic representations (or conceptual nodes; Kroll and Dijkstra, 2002). Further, RHM suggests that while L1 words are directly associated with conceptual nodes, L2 words are weakly linked to conceptual nodes; particularly, L2 words are linked to the conceptual node *via* their L1 equivalents, especially, in bilinguals with lower L2 proficiency. As a result of these asymmetries in word-concept relationships between L1 and L2, RHM predicts that L2-L1 translation or interpreting should be faster than that of L1-L2 at the lexical level (Kroll and Stewart, 1994). RHM also predicts that directionality can be a vital modulator in word translation, together with L2 competence, translation expertise, and the concreteness level of stimuli and cognate status (García, 2015). Among these modulators, directionality is strongly linked to the level of L2 proficiency. However, earlier studies have refuted the hypothesis that L2-L1 translation or interpreting should be faster than L1-L2 translation (Sholl et al., 1995), or translation processes are semantically mediated in both directions irrespective of the levels of L2 proficiency (Duyck and Brysbaert, 2004). Furthermore, several studies on highly proficient L2 subjects (i.e., conference interpreters) found no directionality asymmetry; instead, they observed an L2-L1 advantage among bidirectional interpreters (Chmiel, 2016; Lin et al., 2018). This indicates that the conceptual route may still be more crucially implicated in L1-L2 than in L2-L1 interpreting; theoretically speaking, L2-L1 interpreting at the lexical level is easier than the reverse direction due to weaker links between the speakers’ L2 words and the shared conceptual nodes (Christoffels et al., 2006). As has been mentioned in the previous section, the model is to a large extent based on closely-related European languages. Thus, it remains an empirical question whether previously directionality effects observed in translation/interpreting between closely related European language also apply to typologically distant language pairs such as Chinese and English.

### Directionality Effect in Word Translation

As a window to biligual language processing, lexical translation (or word translation) tasks have been administered in different control settings to investigate the directionality effect on bilinguals and interpreters (García, 2015). Being the primary unit of translation, lexical translation is a necessary step in the interlingual processing of all types of translation activities. Besides, it has been acknowledged as a coping strategy in simultaneous interpreting (SI) when interpreters are under pressure or fatigue (Darò and Fabbro, 1994). Psycholinguistic studies have investigated how bilinguals make lexical decisions in lexical translation by regulating the concreteness level of stimuli (De Groot, 1992) and cognate status (Sáchez-Casas et al., 1992). Overall, it has been confirmed in all these studies that the translation asymmetry effect is supported though seemingly subject to a number of variables such as translation expertise and level of L2 proficiency. Among all the variables, asymmetry effect is most sensitive to L2 proficiency (De Groot, 1992).

Many studies have confirmed that the asymmetry effect appears to decrease when the level of L2 proficiency increases, regardless of other factors in L1-L2 or L2-L1 translation or interpreting (Christoffels et al., 2006; Brysbaert and Duyck, 2010; Tytus, 2017). In this regard, the conceptual mediation route in translation can be used to explain the processing differences in different translation or interpreting directions. Because of the more automatic processing route, the advantage of L2-L1 over L1-L2 word translation weighs on the category of stimulus-level factors, i.e., concreteness level of stimuli (García, 2015), cognate status (Janyan et al., 2009), and phonological similarities (Kim et al., 2018). Although Chaouch-Orozco et al. (2021) attempted to clarify the weight of stimulus-level and individual-level factors in directionality in lexical translation, their findings fall exclusively under the category of stimulus-level factors.

Regarding the investigations on the category of individual-level factors (L2 proficiency, L2 exposure/use, and translation expertise), a few studies have found evidence in support of the RHM hypothesis that the effect of directionality appears among unbalanced bilinguals or L2 learners. For instance, unbalanced bilinguals appear to have developed asymmetric relationships between category names (Chen et al., 2016). Poarch et al. (2015) discovered that fifth-grade Dutch L2 learners were faster in L2-L1 than in L1-L2 translation, which is consistent with RHM. Even for high-proficiency bilinguals, their response time in lexical decision was also facilitated by convert rhymes (Menenti, 2006).

Christoffels et al. (2006) compared lexical retrieval between professional interpreters and that of bilingual university students and highly proficient L2 teachers. The results showed that interpreters outperformed university students in speed and accuracy of lexical retrieval in both directions; however, there were no significant differences between interpreters and L2 teachers using the same measures. Notably, their findings confirmed that the directionality effect only occurs in the student group. Furthermore, García et al. (2014) found no directionality effect in word translation among beginner translation students, advanced translation students, and professional translators. These studies demonstrate that translation expertise, similar to L2 proficiency level, is directionality independent.

The asymmetry is also supported by behavioral and neuroscientific observations. Behaviorally, Jost et al. (2018) found slower response times for L1-L2 lexical translation compared to translation of L2-L1 direction. On the electrophysiological level, stronger activation was noted during L1-L2 translation compared to L2-L1 translation at about 200ms after word presentation, showing that L2-L1 translation is faster than that of L1-L2 in reaction time. In a functional magnetic resonance imaging (fMRI) study, Zheng et al. (2020) revealed that L2-L1 lexical translation involves increased functional activity between a core semantic hub (the left anterior temporal lobe) and key nodes of attentional and vigilance networks (left inferior frontal, left orbitofrontal, and bilateral parietal clusters). Thus, this indicates that the advantage of L2-L1 translation appears to involve enhanced coupling between semantic and attentional mechanisms. This suggests that asymmetries in cross-language processing mirror the dynamic interactions between linguistic and domain-general systems.

### Directionality Effect in Translation and Interpreting Activities

Translation and interpreting studies have explored the directionality effect under different settings, including regular translation practices (Hunziker Heeb, 2016; Pokorn et al., 2020), and interpreting practices including CI (Chen, 2020), SI (Rayaa, 2017; Lin et al., 2018), sight interpreting (Yuan and Wang, 2019), and even signed language interpreting (Nicodemus and Emmorey, 2013; Wang and Napier, 2015). As has been stated in the previous section, the category of stimuli-level factors has been thoroughly investigated at the lexical level, specifically the concreteness of stimuli and cognate status. This line of research has begun to adopt near-naturalistic texts as source input in CI and SI tasks to investigate the mechanisms whereby the category of individual-level factors interact with the directionality effect. A considerable number of studies recruited only professional interpreters (Wang and Napier, 2015; Chmiel, 2016; Hunziker Heeb, 2016; Chen, 2020). Meanwhile, others investigated the mechanisms by which novice interpreters may perform in different translation directions (Pavlović, 2007; Lin et al., 2018; Pokorn et al., 2020). Besides, a novice-expert paradigm was also applied to assess how the two groups differed in their performance under the influence of directionality (Nicodemus and Emmorey, 2013).

With the aid of pen recording and eye-tracking techniques, Chen (2020) studied how professional interpreters performed CI tasks in two directions and discovered that the professional interpreters appeared to use more full words (compared to abbreviations) in notes for L2-L1 interpreting than for the other direction. This finding suggests that interpreters are under a higher level of cognitive load during L2-L1 interpreting, and even professional interpreters are still subject to the directionality effect. However, the effect of directionality disappeared among professional signers when they interpreted between English and Australian Sign Language (Wang and Napier, 2015). Even among interpreters themselves, there is a difference in opinion regarding directionality effects. Pavlović (2007) conducted a questionnaire survey among professional translators and interpreters in Croatia and found that 45% rated themselves with better performance in L1-L2 translating and interpreting than the reverse direction.

Among novice interpreters, a number of studies on directionality effects found support for an L2-L1 advantage in interpreting. Yuan and Wang (2019) research on novice interpreters identified a directionality effect on fluency, with fewer speech repairs and pauses in L2-L1 sight interpreting than in the other direction. Lin et al. (2018) found that working memory and directionality yield a significant effect on fluency among novice interpreters. Moreover, it has also been found that interpreters’ direction preference and interpreting performance are not positively correlated (Nicodemus and Emmorey, 2013). Nevertheless, Pokorn et al. (2020) found that factors including L2 proficiency and the encyclopedic knowledge of the interpreter play a bigger role in affecting the interpreting quality than directionality among a group of novice interpreters.

With regard to the language pair of Chinese (L1) and English (L2), participants of such studies include both professional interpreters (Chang, 2005; Chang and Schallert, 2007) and student interpreters (Lin et al., 2018; Chen, 2020).

In terms of individual factors, studies have found that the difference in professional interpreters’ performance appears to be the result of a combination of factors rather than just directionality, such as discourse structures and audience expectations (Chang, 2005; Chang and Schallert, 2007). However, directionality yields a significant effect on fluency, with better fluency in L2-L1 SI than in L1-L2 SI (Lin et al., 2018). The same effect is also observed in L2-L1 CI which is more fluent but less accurate than the other direction (Chen, 2020). To further probe into the unequal cognitive efforts between L1-L2 with L2 to L1 interpreting, He et al. (2021) adopted functional near-infrared spectroscopy (fNIRS) to study two groups of interpreters (one group with high interpreting expertise and another group with low interpreting expertise). They found that the directionality effect exists in both groups though both groups display different brain activities. Although directionality effect is found to work to different extent in both European language pairs and more distance language pairs such as Chinese and English, no research has attempted to examine how language pair can be a variable in affecting the magnitude of directionality effect. In other words, the question of directionality and language distance raised by Malkiel (2004) remains largely unexplored. After reviewing the state-of-the-art of directionality research in the field of interpreting studies, we found that although there exists a number of studies that explored the directionality effect, research that has applied a more focused lens to qualitatively explore directionality effect involving student interpreters are rare.

In summary, regarding the impact of the individual-level factors on directionality, the studies have reported conflicting results. It remains unclear whether interpreters perform differently in different interpreting directions and to what extent directionality interacts with other factors including language proficiency, working memory, and translation expertise to make an impact on the interpreting performance.

When considering stimuli-related variables, as per RHM and relevant studies testing the RHM hypothesis, word translation is found to be modulated by directionality; these stimuli-related effects may disappear as L2 proficiency increases, suggesting that the conceptual level has a role in both directions, while the lexical routes appear to operate asymmetrically (see García, 2015, for a review). In view of the limitations of existing studies, it is worth investigating whether the direction-dependent asymmetry in lexical interpreting also applies to a different context with more naturalistic stimuli. Previous studies have identified the attenuation of directionality effects with an increase of L2 proficiency and translation expertise (note that all these studies are based on European languages), it is worthwhile to examine how a group of trainee interpreters with Chinese L1 and English L2 might perform in interpreting tasks of different directions. In this study, we recorded the L1-L2 and L2-L1 CI of 66 subjects in a national interpreting contest. Then, recordings and transcripts were analyzed with an enhanced protocol of CI quality (see section “The Enhanced Protocol of CI Quality”). Contestants were also asked to fill the Translation and Interpreting Competence Questionnaire (TICQ) designed by Schaeffer et al. (2019) to provide demographic information in language learning, and hours of training in translation and interpreting. This study has two objectives:

1. In order to investigate to what extent directionality effect can make an impact on different facets of interpreting quality, a multi-dimensional approach consisting of various measures will be used to look into the different aspects of interpreting quality, including content, form, and delivery (Zwischenberger, 2010).
2. Regarding individual-related variables, we aimed to evaluate whether entry-level novice interpreters would be influenced by directionality, i.e., whether the L2-L1 advantage in directionality would also be effective among novice interpreters and how that may modulate the performance of novice interpreters.

## The Enhanced Protocol of CI Quality

### Interpreting Quality: A Review

Interpreting involves extracting meaning in one language then expressing it in another language; hence, interpreting quality may be affected by an array of factors and it is difficult to use one single scale to assess all interpreting events (Zwischenberger, 2010). Interpreting assessment has been a central topic and raised significant interest among scholars and trainers of interpreting (Lee, 2015). Studies have proposed different assessment systems for CI. After reviewing existing literature on interpreting performance assessment, Lee (2015) identified three major categories for assessing interpreting performances, i.e., “content,” “form,” and “delivery,” following Zwischenberger (2010) assessment model. Table 1 summarizes major studies on assessing the quality of interpreting.

**Table 1:** Overview of studies on CI quality.

Researcher	Assessment criteria
Roberts (2000)	(i) Understanding, (ii) accurate rendition of ideas in the target language, (iii) handling of names and numbers, and (iv) appropriate target language expression
Clifford (2001)	Deixis, modality, and speech acts
Pöchhacker (2001)	(i) Accurate rendition, (ii) adequate target language expression, (iii) equivalent intended effect, and (iv) successful communicative interaction
Lee (2008)	(i) Accuracy (deviations, intended effects, faithful rendering, coherence, and logic), (ii) target language (TL) quality (grammaticality, phonology, morphology, syntax, naturalness, register, style), and (iii) delivery (articulation, pause, hesitation, false starts, fillers, excessive repairs, frequent self-corrections, voice, etc.).
Zwischenberger (2010)	(i) Content (sense consistency with the original, logical cohesion, completeness), (ii) form (correct terminology, correct grammar, appropriate style), and (iii) delivery (fluency of delivery, lively intonation, pleasant voice, synchronicity, native accent)
Liu (2013)	(i) Accuracy and (ii) delivery (hesitation, repetition, self-correction, redundancy, usages of grammar or terms, coherence)
Choi (2013)	(i) Accuracy (opposite sense, false sense, no sense, imprecision), (ii) expression (terminological error, lexical error, grammatical error), and (iii) presentation (speed error, overuse of pause fillers/backtracking)
Zhao and Dong (2013)	(i) Accuracy, (ii) delivery, and (iii) strategy and manner.

### The Enhanced Protocol of CI Quality

In their research to investigate whether the use of digital voice recorder would help yield better results than the conventional CI methods, Hamidi and Pöchhacker (2007) specifically proposed a transcript-based protocol in the assessment of CI performance (see Table 2).

**Table 2:** Hamidi and Pöchhacker (2007) CI quality protocol.

1. Fluency: length and speed	Duration (seconds): length of the recording Number of words: from the transcript Speech rate: words per minute
2. Pauses	Number of pauses Percentage of total duration Number of long pauses (> 1.5s) Longest pause Number of filled pauses (voiced hesitations)
3. Source-target correspondence: three types of deviations	Two levels of omission Addition Substitution
4. Quality of expression	Grammatical errors Syntactical errors Lexical errors False starts Repetitions Slips of the tongue Reformulations

Our study made reference to the model by Hamidi and Pöchhacker (2007) and also adopted a standard information recall measure, which captures coarse-grained processes encompassing listening comprehension, higher-order cognitive processing, and linguistic reformulation skills (Çakmak and Erçetin, 2018; Vander Beken et al., 2020). This measure also fits into the category of “content” in Zwischenberger (2010). Following the standard assessment procedures (Roediger and Karpicke, 2006; Vander Beken and Brysbaert, 2018; Vander Beken et al., 2020), the presence and correctness of ideas were also scored in each transcription. Idea units were given one point if correctly recalled, 0.5 points if partially recalled, and 0 points if incorrectly recalled or omitted. Thereafter, the proportion of correctly recalled information was calculated over the total number of idea units in each segment.

Considering the different generic features between Chinese and English, the model of Hamidi and Pöchhacker (2007) was slightly modified to suit the current study. In the end, the category of delivery includes measures including pauses, false starts, repetitions and slips of tongue, whereas “quality of expression” includes false collocation, missing independent clauses and redundancy, which was based on previous research (Ting et al., 2010). The modified enhanced CI quality protocol used in the current study is shown in Table 3.

**Table 3:** The enhanced CI quality protocol.

1. Content (information completeness)	
2. Fluency	Duration (seconds): length of the recording Number of words: from the transcript Speech rate: word per minute
3. Delivery	Number of filled pauses Number of unfilled pauses False starts (interruption of a sentence followed by another complete sentence with a change in meaning) Repetitions (unwarranted reiteration of a word or a phrase, usually after a pause) Slips of tongue (deviations from the intended form of an utterance)
4. Quality of expression	False collocation (wrong choice of word and collocation) Missing independent clause (IC) Redundancy (information has been iterated, semantic redundancy in particular)

Regarding the evaluation of information completeness, we followed previous practice in which the idea units in each source transcript were identified using standard procedures (Mills et al., 1993). Next, we followed the validated scoring protocols used in previous studies (Karpicke and Roediger, 2010; Blunt and Karpicke, 2014) to score the units. Each unit was given 1 point if correctly recalled, 0.5 points if partially recalled, and 0 points if incorrectly recalled or omitted. Illustrations of the metrics of delivery and quality of expression are seen in Table 4.

**Table 4:** Illustration of the metrics of delivery and quality of expression.

3 (a). Number of filled pauses	Number of fillers in the transcript
3 (b). Number of unfilled pauses	Number of unfilled pauses >1.5s
3 (c). False starts	嗯//如今//嗯嗯//现在让我来告诉大家有关海洋酸化的严峻现实// *Um//nowadays//um, um//Now let me tell you about ocean acidification, a serious reality* (The sentence began with a false start “nowadays,” then changed to “now.”)
3 (d). Repetitions	现在疾病可以在二十四二十四小时之内传播到世界各地的角落里 *Now a disease can spread to every corner of the world in twenty-four twenty four hours* (There are two “twenty four” in the sentence.)
3 (e). Slips of tongue	海洋占据了我们星球的百分三分之二的面积 *The ocean covers percentage two thirds of our planet* (The word “percentage” is a slip of tongue.)
4 (a). False collocation	人们更容易被流感传播 *people tend to be transmitted by flu* (传播 “transmit” is a wrong collocation with 人们 “people” in Chinese.)
4 (b). Missing IC	这也是导致流行性疾病 *This also causes epidemics…* (Independent clause is missing in the above fragment.)
4 (c). Redundancy	现在我要说一个非常严重的现象,是关于海洋酸化这一现象 *Now I would like to say a serious phenomenon, it’s about ocean acidification, this phenomenon* (The expression “*this phenomenon*” is a redundancy.)

In terms of the distinction between 3 (delivery) and 4 (quality of expression), as illustrated in Table 4, the measurement of “delivery” stresses the level of fluency with some disfluency indices such as pauses, fillers, and false starts. The measurement of “quality of expression” focuses on interpreting quality by examining the grammatical mistakes such as false collocation, incomplete sentences, and redundant expressions.

Two independent raters separately scored all the enhanced CI quality protocols listed in Table 3. A third rater (the first author) resolved all discrepancies to reach an agreement. The inter-rater agreement was 97% of information units for the L2-L1 task and 93% for the L1-L2 task. The remaining respective 3 and 7% of the units were resolved in a three-way discussion between both raters and the third rater, following reported procedures of previous studies (Karpicke and Roediger, 2010; Blunt and Karpicke, 2014).

## Methods and Results

### Participants

The participants are 66 undergraduate and postgraduate students from translation and interpreting programs of various universities in Southwest China (note that these participants are attending a regional interpreting competition). All participants were native Chinese speakers who learned English through formal education. Their ages ranged from 20 to 26years. Notably, they were right-handed and none of them had neurological or psychiatric antecedents. Table 5 shows the demographic and language profile of the participants based on Schaeffer et al. (2019).

**Table 5:** Demographic and language profile of subjects (mean and SD).

Demographic data	
Sex (F: M)	55: 11
Years of age	24.08 (1.45)
Years of education	16.45 (1.36)
**Linguistic profile**	
Age of L2 learning	8.86 (2.32)
L1 competence^*^	80.72 (10.53)
L2 competence	66.25 (10.36)
Competence in L1-L2 CI	63.34 (13.20)
Competence in L2-L1 CI	62.19 (12.92)
Weekly dedication to L1-L2 CI (hours)	3.90 (3.82)
Weekly dedication to L2-L1 CI (hours)	4.21 (2.87)
Competence in L1-L2 translation	63.12 (12.33)
Competence in L2-L1 translation	64.43 (12.07)
Weekly dedication to L1-L2 translation (hours)	3.87 (2.57)
Weekly dedication to L2-L1 translation (hours)	4.66 (2.76)

* For the questions on competence, students were asked to rate their competence based on a 100-point scale.

### Materials and Procedure

The materials are eight recordings (three from L1-L2 and five from L2-L1) in the second round of the nationwide interpreting competition. Each participant was required to perform CI in two directions. The first task is from L2 to L1 and participants were randomly assigned a passage, then followed by a L1 to L2 task with a passage which is also randomly assigned from the database. The distribution of passages is given in Table 6.

**Table 6:** The distribution of passages.

L2-L1	L1-L2
“Ocean acidification”	“Autism”
“Pandemic”	“e-Sports”
“Gig economy”	“Loneliness”
	“Aging population”
	“Chinese diplomacy”

They were transcribed by the authors and the minimal instances that required editing were resolved through consensus between the first and the second authors. Transcriptions were saved as DOC files for further processing and analysis. Details of the eight recordings are provided in Table 7.

**Table 7:** Mean and SD (in parentheses) of source recordings.

	L1-L2	L2-L1
Duration (seconds)	73.00 (2.94)	63.00 (3.39)
Number of words	143.00 (14.45)	206.40 (7.44)
Speech rate (word/second)	3.20 (0.56)	1.53 (0.33)

Participants remotely accessed the recordings through their computers using a headset. They could listen to the recordings once only and then required to interpret the task once the recording finished. The first was an L2-L1 CI task. Participants were required to listen to an English passage randomly assigned from the five topics. There was no stop in the recording and students were allowed to freely take notes. Afterwards, the participants were asked to interpret the English passage into Chinese right after the recording stops. The platform started to record participants’ voice once the source recording stopped. Recording of the interpreting output would last 1.2 times of the original recording before it stopped. The L2-L1 task was then administered upon completion of the L1-L2 task, which was also randomly assigned to participants from the three topics mentioned above. The same procedure was repeated as in task 1. Recordings which contain students’ interpreting output that fall within the allowed time window were regarded as eligible in the contest.

Recordings were first automatically transcribed through iFlytek, a software reported to have 97.5% accuracy in English recognition (Wang et al., 2018). Subsequently, three individual copyeditors checked each transcribed file against the recording to ensure optimal quality. Discrepancies in transcriptions were resolved by consensus among all three copyeditors. Based on the model proposed by Hamidi and Pöchhacker (2007), we first calculated the mean frequency of filled pauses (meaningless vocalizations like “um” between utterances) and unfilled pauses (a pause not “filled” by a hesitation form) in each recording. All annotations were marked with Adobe Audition (version 13). We then calculated the mean frequency of three disfluency metrics in each recording.

First, delivery was measured in terms of quantifying pauses and disfluencies. Based on the model proposed by Hamidi and Pöchhacker (2007), we first calculated the mean frequency of filled pauses and unfilled pauses in each recording. All annotations were marked with Adobe Audition (Version 13). Then, the mean frequency of three disfluency metrics was calculated in each recording: false starts (interruption of a sentence followed by another complete sentence with a change in meaning), repetitions (unwarranted reiteration of a word or a phrase, usually after a pause), and slips of the tongue (deviations from the intended form of an utterance).

In addition, the quality of expression in terms of grammatical, syntactic, and lexical errors was measured (Hamidi and Pöchhacker, 2007). In line with previous research (Ting et al., 2010), the following variables were also taken into consideration: misinformation (use of wrong word forms or structures), wrong sentence structure [lack or misuse of a subject and/or a finite (+tense) verb, and/or an independent clause], and wrong word selection (non-native-like word combinations).

## Results

Experimental data and analytical scripts are publicly available on Open Science Framework.^1^ Paired samples *t*-tests were used to compare the two directions of interpreting. Cohen’s *d* was used to indicate the effect sizes and classified as small (*d*<0.2), medium (*d*<0.5), and large (*d*<0.8). As described in section “Directionality Effect in Translation and Interpreting Activities,” ten metrics were measured in four categories, i.e., speech rate, information completeness, delivery, and quality of expression. Their descriptive statistics are detailed in Table 8.

**Table 8:** Mean and SD (in parentheses) for all measures in CI tasks, with *t*-tests between directions.

Measures		L2–L1 task	L1–L2 task	Between-direction *t*-test
Speech rate		3.20 (0.55)	1.54 (0.33)	*t* (65)=30.886, *p* <0.001
Information completeness		0.70 (0.17)	0.67 (0.14)	*t* (65)=1.422, *p* =0.159
Delivery	Unfilled pauses	2.00 (3.11)	2.70 (4.88)	*t* (65)=−1.548, *p* =0.077
	Filled pauses	1.48 (2.92)	2.03 (3.63)	*t* (65)=−1.796, *p* =0.126
	False starts	0.35 (0.91)	0.11 (0.40)	*t* (65)=2.014, *p=* 0.048
	Repetition	0.97 (1.35)	1.45 (1.66)	*t* (65)=−2.216, *p* =0.030
	Slips of tongue	0.14 (0.42)	1.23 (1.44)	*t* (65)=−3.701, *p* <0.001
Quality of expression	False collocation	1.35 (1.13)	3.80 (2.60)	*t* (65)=−7.172, *p* <0.001
	Missing IC	0.14 (0.42)	1.23 (1.44)	*t* (65)=−5.559, *p* <0.001
	Redundancy	0.62 (1.33)	2.12 (1.93)	*t* (65)=−5.629, *p* <0.001

**The speech rate** in the L2-L1 task was significantly faster than that in the L1-L2 task [*t* (65)=30.886, *p*<0.001, *d*=0.66]. No effect of directionality was found regarding **information completeness** [*t* (65)=1.422, *p*=0.159, *d*=0.26]. There was an effect of directionality in three metrics of **delivery**, namely false starts [*t* (65)=2.014, *p*<0.048, *d*=0.33], repetition [*t* (65)=−2.216, *p*<0.030, *d*=0.35] and slips of tongue [*t* (65)=−3.701, *p*<0.001, *d*=0.66]. The directionality effect was also found in Quality of Expression in all three metrics, namely, false collocation [*t* (65)=−7.172, *p*<0.001, *d*=1.24], Missing IC [*t* (65)=−5.559, *p*<0.001, *d*=0.99], and Redundancy [*t* (65)=−5.629, *p*<0.001, *d*=0.88]. Overall, we can see that directionality effect holds true in the majority of metrics used in this study.

## Discussion and Conclusion

This work evaluated the impact of directionality on the CI performance of trainee interpreters, with L1 in Chinese and L2 in English. Based on the enhanced CI quality scale of Hamidi and Pöchhacker (2007), we examined if the CI interpreting quality varies between L1-L2 and L2-L1 interpreting tasks. The enhanced CI quality model has four categories, including speech rate, information completeness, delivery, and quality of expression. We observed significant differences in speech rate, delivery, and quality of expression between L1-L2 and L2-L1 interpreting; no significant difference was found in information completeness. These findings suggest that directionality affects the performance of trainee interpreters.

For speech rate, we found a significant difference between the two interpreting directions, with the rate of L2-L1 interpreting faster than that of L1-L2. This result echoed the findings of Chen (2020), who studied professional interpreters with the same language pairs (L1 in Chinese and L2 in English). Our results showed that speech rate is a directionality-dependent variable, regardless of language proficiency and translation expertise. Nonetheless, it should also be noted that speech rates of native and non-native speakers vary considerably. Studies have shown that the average speaking rate is slower in non-native than in native speakers because of suprasegmental differences between the speakers’ L1 and L2 languages (Trofimovich and Baker, 2006). Specifically, interpreters with Mandarin Chinese as L1 and English as L2 tend to produce faster speech rate in Mandarin Chinese than in English (Chen and Robb, 2004). Therefore, the differences in speech rate between the two directions might be due to the language distance of source language and target language instead of translation directions.

Regarding information completeness of CI, information retention often requires multitasking, including listening comprehension, high-order cognitive processing, and linguistic reformulation skills (Çakmak and Erçetin, 2018; Vander Beken et al., 2020). Since we did not identify significant differences between the two directions, it possibly implies that regardless of the interpreting from or into a native language, the strength of the memory trace in both L1 and L2 remains at the same level. That is, the amount of information retained from cross-language reformulation involves the mechanisms by which information is encoded and retrieved in both L1 and L2. In the L2-L1 CI task, the information was formulated in the L2 of the subjects and they were required to retrieve and decode the same information into L1. In contrast, in the L1-L2 CI task, information in L1 must be represented in L2. Vander Beken and Brysbaert (2018) probed into how information encoding and retrieval differ between materials studied in first and second language (L1 and L2) and found that subjects performed at the same level on the recognition test in both languages with cued recalls. Furthermore, an investigation on whether unbalanced bilinguals recall study materials in L2 as well as in L1, despite an L2 disadvantage in recalling short texts, found no such disadvantage in true/false recognition test (Vander Beken et al., 2020). As documented by Vander Beken et al. (2020), when reading or listening to a text for a subsequent memory or code-switching test, it is possible to largely translate the information into language-independent, and abstract memory codes.

As for delivery, significant differences were found between the two directions. In this category, we captured the frequency of filled and unfilled pauses together with disfluency features (i.e., false starts, repetitions, slips of tongue); notably, each has been included in several quality interpretation assessment scales as critical values in delivery (Lee, 2008; Choi, 2013; Liu, 2013). According to descriptive statistics in Table 8, except for the “false starts,” the other four metrics recorded a higher frequency in the L1-L2 than that in the L2-L1 task. Scholars unanimously agree that pauses and disfluencies are cognitive overloading traces of interpreters and reflect the complexity of source speech and its information load (Bóna and Bakti, 2020). Moreover, Gile and Chai (2009) listed some linguistic and prosodic features in the source speech which may cause an increase in the processing capacity of interpreters. These features are considered cognitive problem triggers. Once these triggers appear, the required overall processing load may exceed the overall cognitive load of the interpreter when disfluencies and pauses may be produced. Since all nine source speeches were provided by the contest committee for the interpreting contest, the difficulty level is assumed to be similar, meaning that the variable of triggers was well controlled. This was confirmed by the mean length and SD of the source recordings (see Table 7). As a result, such differences in delivery can be attributed to the directionality effect.

As a major metric in delivery, pauses (filled and unfilled) have been used in various studies to evaluate the effect of directionality and among different subjects (novice or expert). Our findings of no significant differences in both filled and unfilled pauses between the two interpreting directions are consistent with the findings reported in previous studies. For example, Lin et al. (2018) found novice interpreters exhibited similar patterns in both filled and unfilled pauses, with slightly more instances in the L1-L2 CI task than in L2-L1. Chen (2020) also identified similar results with professional interpreters regarding filled and unfilled pauses. On the other hand, the other three metrics (i.e., false starts, repetitions and slips of tongue) of the “delivery” category exhibited significant differences between the two interpreting directions. This may suggest that the impact of directionality on delivery is prominent, irrespective of the language proficiency and translation experience of interpreters.

Similarly, we observed the impact of directionality in the category of quality of expression. To our knowledge, this is the first study exploring whether the quality of expression in CI is susceptible to the directionality effect. This category, which consists of three metrics, i.e., false collocation, missing independent clause, and redundancy, primarily reflects the manner in which grammatical mistakes occur in interpreting output. The metrics used in this category have been widely employed to assess the interpreting performance in various contexts (Zwischenberger, 2010; Choi, 2013; Liu, 2013). Our findings indicate that trainee interpreters appear to make more grammatical mistakes and other types of reformulation-related errors in L1-L2 CI than in the other direction. Considering that the metrics in this category are more relevant to language forms instead of language content, the results suggest that students might be weaker in their L2 proficiency than expected. As has been stated in the review earlier, the impact of directionality attenuates with the L2 proficiency level using lexical-level stimuli.

Taking together the significant effects of directionality on delivery and quality of expression, the CI performance of trainee interpreters was better in the L2-L1 than in L1-L2 task. Studies have attributed several factors to the impact of directionality, including market and speech characteristics (Fernández, 2005), interpreting mode (Nicodemus and Emmorey, 2013), language pair (Padilla, 2005), audience features, and the discourse structure of the working language (Chang and Schallert, 2007). However, almost all these studies often work with highly controlled lexical stimulus of different concreteness level and cognate status. In contrast, our study is based on a lifelike CI task (i.e., interpreting a natural passage in two directions instead of lexical translation). Our results have affirmed the findings of previous studies which are based on lexical translation that the L2-L1 advantage also exists in interpreting natural text. This study confirms the RHM hypothesis by supplying further evidence from text-level interpreting.

Delivery and quality of expression tend to gauge how target language reformulation is influenced by directionality. Interpreting is a complex cognitive activity involving an array of interwined sub-cognitive tasks. Notably, it is impossible to link any sub-tasks to the final product of interpreting. In bilingual processing, it is generally acknowledged that one language must be inhabited when producing the other. The act of interpreting requires constant and repeated activation as well as inhabitation of one language or the other. Thus, the cognitive mechanism underlying delivery and quality of expression is more inclined towards L2 production, but likely to be inhibited by L1 among unbalanced bilinguals (Declerck et al., 2019). Our study suggests that L1-L2 interpreting involves stronger inhibition of L2 than L2-L1 interpreting involves inhibition of L1. The evidence to support such a claim can be found in the number of mistakes in grammar and disfluencies in either direction. From the perspective of information retention and the general phases of CI as stated above, the information received in L1 or L2 is possibly transformed into a language-independent memory code in any cross-linguistic language processing (Vander Beken and Brysbaert, 2018; Vander Beken et al., 2020). In this case, the completeness level of information may be unaffected by the direction of translation, but other factors such as L2 proficiency and memory span warrant further investigation.

Our study is not without limitations. First, our data were derived from interpreting tasks conducted remotely *via* an online interpreting platform. Such a setting, despite its near-naturalness in terms of environmental setup and interpreting materials, can give rise to some issues that might confound the study results. For example, while participants were allowed to take notes during interpreting, such notes were inaccessible to the researchers. We are fully aware that note-taking can be a key modulator in interpreters’ performances. To what extent note-taking can affect the directionality effect is worthy of future investigations In addition, the language pair used in the current study (L1: Chinese and L2: English) consists of two languages that are genetically different from each other. Results of the current study indicate that L2-L1 advantage appeared with novice interpreters in conducting CI tasks in speech rate, quality of expression, and some delivery metrics including false starts, repetition and slips of tongue. This is in line with Yuan and Wang (2019) findings that there were fewer disfluencies in sight translation from L2 to L1 than the other way around. However, our findings do not corroborate studies involving closer language pairs with novice interpreters as participants, where no directionality effect was found (Nicodemus and Emmorey, 2013; Pokorn et al., 2020). Finally, the order of the interpreting tasks in the two directions was not counterbalanced in our study; that is, participants always did L2-L1 interpreting task first then followed by the L1-L2 interpreting task, as per the requirements of the nation-wide interpreting competition. Future studies might need to adopt a more balanced design to control the confounding variable of task sequence.

The findings have great pedagogical implications. The notion that translating from one’s mother tongue into a foreign language does not have much value is deeply rooted in the western world, as evidenced by the mainstream practice by international organizations which accept only L2-L1 translation (Pavlović, 2013). In direct contrast to the European practice which emphasizes “direct translation” where translators or interpreters normally work from L2 to L1, “inverse translation” where translators and interpreters work from L1 to L2 is prevalent in China and becoming even more widespread (Liu and Afzaal, 2021). In this regard, L1-L2 interpreting has an important role to play in the interpreting classroom. Our research findings that L1-L2 interpreting is more challenging (e.g., more disfluencies, more grammatical, and collocational mistakes) than the reverse direction for novice interpreters due to the directionality effect has implications for interpreting pedagogy. In view of the findings and widespread L1-L2 interpreting in China, we suggest that interpreting teachers can adopt different teaching approaches by paying more attention to some aspects unique to L1-L2 interpreting.

In conclusion, we explored how the performance of student interpreters is influenced by directionality using naturalistic data from an interpreting contest. Our research has provided insights from the perspective of CI between Chinese and English, two languages that are typologically different from each other. Our findings are broadly consistent with previous findings based on different language pairs, and with previous findings based on participants of different language proficiency and professional experience (Lin et al., 2018; Chen, 2020). We have shown that the surface language “form” in CI quality is more sensitive to directionality than language “content.” These findings have yielded some new insights into the role of directionality in interpreting and provided pedagogical insights for interpreter training. However, the findings should be further validated with the novice-expert paradigm with a more vigorous control on demographic backgrounds, language proficiency in both L1 and L2, interpreting competence, and cognitive capacity.

## Data Availability Statement

The datasets presented in this study can be found in online repositories. The names of the repository/repositories and accession number(s) can be found at: https://osf.io/su8zy/.

## Ethics Statement

The studies involving human participants were reviewed and approved by University of Electronic Science and Technology of China. The patients/participants provided their written informed consent to participate in this study.

## Author Contributions

IC: conceptualization, methodology, formal analysis, investigation, resources, writing – original draft, and writing – review and editing. KL: writing – original draft and writing – review and editing. NZ: writing – review and editing and formal analysis. All authors contributed to the article and approved the submitted version.

## Funding

This work was supported by the Fundamental Research Funds for the Central Universities, UESTC (grant no.: ZYGX2019J140).

## Conflict of Interest

The authors declare that the research was conducted in the absence of any commercial or financial relationships that could be construed as a potential conflict of interest.

## Publisher’s Note

All claims expressed in this article are solely those of the authors and do not necessarily represent those of their affiliated organizations, or those of the publisher, the editors and the reviewers. Any product that may be evaluated in this article, or claim that may be made by its manufacturer, is not guaranteed or endorsed by the publisher.

## Footnotes

^1^ https://osf.io/su8zy/
